# A Case of Calcified Metastatic Colorectal Adenocarcinoma Mimicking a Benign Lesion: Pitfalls in Diagnosis

**DOI:** 10.1155/2015/936260

**Published:** 2015-02-05

**Authors:** Peter Michail, Iftah Amith, Sanila George, Mathew K. George

**Affiliations:** ^1^School of Medicine and Public Health, University of Newcastle, University Drive, Callaghan, NSW 2308, Australia; ^2^University of New England, Elm Avenue, Armidale, NSW 2351, Australia; ^3^Hunter Imaging, 7 Bligh Street, Tamworth, NSW 2348, Australia; ^4^Department of Medical Oncology, North West Cancer Centre, 31 Dean Street, Tamworth, NSW 2340, Australia

## Abstract

The radiological finding of a calcified intracranial lesion commonly represents a slow growing benign mass. Brain metastases originating from colorectal cancers are rare, occurring in approximately 2-3% of patients. Therefore the presence of a calcified brain lesion in a patient with a positive oncological history requires a high index of suspicion for brain metastases. Presented herein is a case of a frontoparietal calcified lesion initially overlooked as a benign tumour. Subsequent imaging following a neurological episode revealed a significant increase in size of the lesion with surrounding tissue oedema, prompting further investigation for suspicion of a calcified metastatic colorectal adenocarcinoma.

## 1. Introduction

The radiological findings of calcified intracranial lesions generally represent slow growing, benign masses [1]. Most common differential diagnoses include angiomas, gliomas, meningiomas, and granulomatous lesions [1]. It is rare for metastatic brain lesions to present with calcification; however these represent approximately 1–3.5% of cases found on computer tomography (CT) [1–4].

We present a case of a frontoparietal calcified brain lesion initially diagnosed as either a meningioma or a granuloma. Subsequently, with a delay of just under a year, the lesion was correctly diagnosed as metastatic colorectal adenocarcinoma.

## 2. Case Presentation

The patient is a 61-year-old female who was diagnosed with colorectal carcinoma (CRC) in 2011. The diagnosis was made after colonoscopic examination revealed an obstructing mass. The patient underwent an anterior resection with subsequent investigations revealing high grade metastatic adenocarcinoma. The patient was started on palliative chemotherapy consisting of FOLFOX and Avastin.

**Figure 1:**
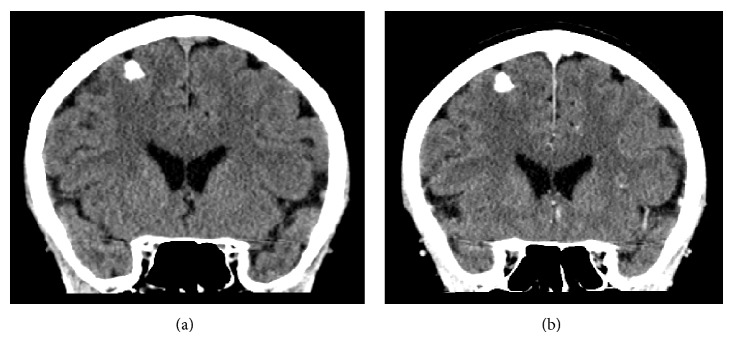
(a) 2013 precontrast CT coronal view. (b) 2013 postcontrast CT coronal view.

Two years after diagnosis (04/2013) a CT brain with contrast was performed revealing an incidental finding of a calcified lesion (Figure 1). The calcified focus was 6 × 10 mm located superficially in the high right frontoparietal region with no obvious enhancement of soft tissue density. It was reported as a diagnosis of either an old meningioma or granuloma. Based on the small size, location, calcification, and uniform enhancement after contrast administration, with no oedema or tissue enhancement, the decision was made not to investigate further and treat conservatively.

**Figure 2:**
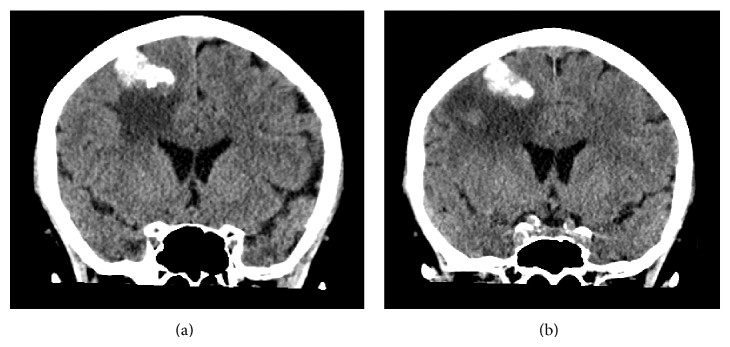
(a) 2014 precontrast CT, coronal view. (b) 2014 postcontrast CT, coronal view.

One year later (March 2014) the patient had an unwitnessed episode consisting of uncontrolled neuromotor disturbances with opthalmoplegia. The event was suspicious for an acute dystonic reaction due to frequent Maxolon (metoclopramide) administration. The episode subsequently self-resolved; however residual left arm weakness was apparent for the following two weeks. A CT brain was organised to exclude metastases and other organic pathologies. The previous calcified lesion on CT was noted to have increased significantly in size to 3 cm with prominent surrounding tissue oedema (Figure 2).

**Figure 3:**
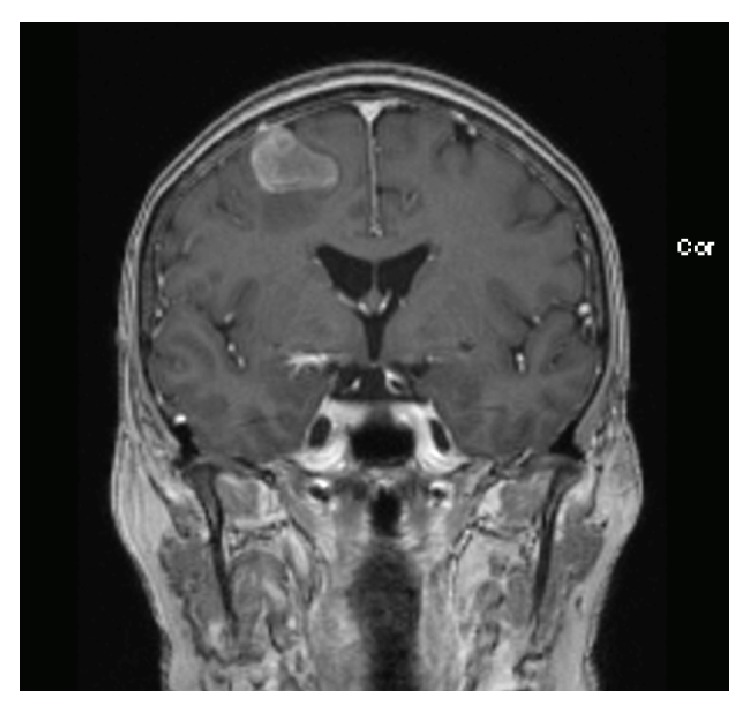
2014 MR image, coronal view.

This was increasingly suspicious of a metastatic brain lesion. MRI scan was organised for confirmation, revealing a nonenhancing intra-axial mass lesion with surrounding vasogenic oedema consistent with the calcified lesion seen on CT (Figure 3). The appearance was not typical for a meningioma, and given the previous history of CRC, it was highly suspicious of a calcified metastatic focus.

**Figure 4:**
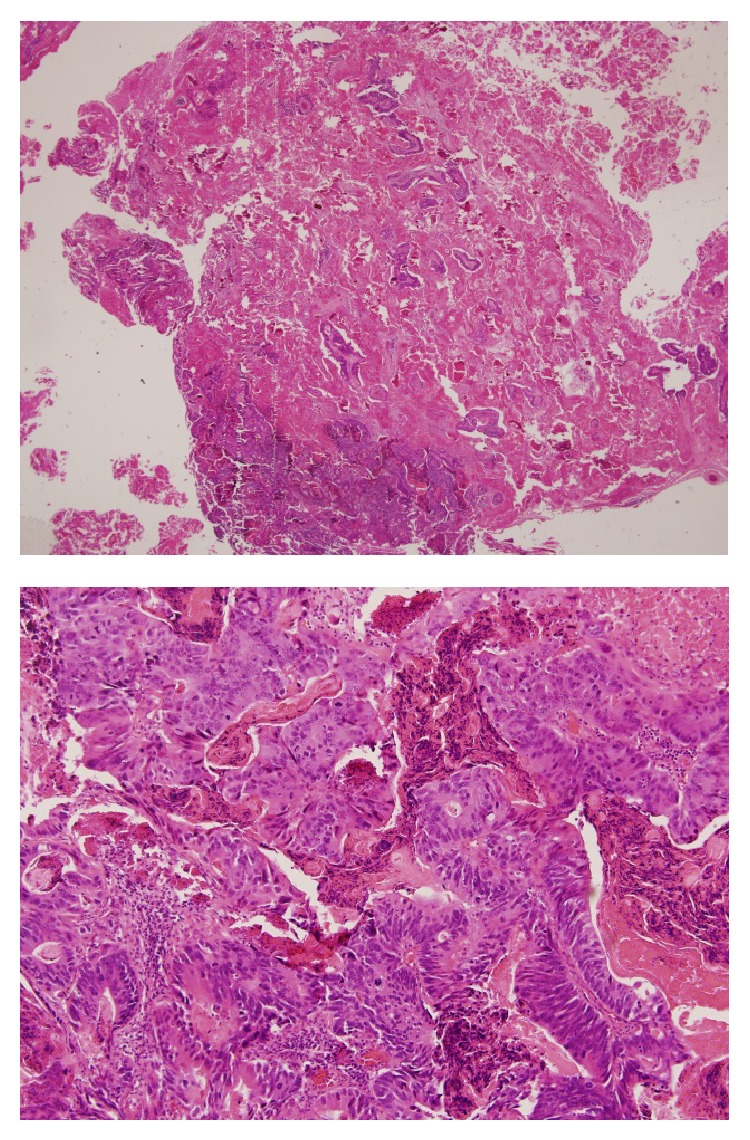
Advanced metastatic adenocarcinoma with focal haemorrhage and palisading glands with papillary formation.

Stereotactic craniotomy was performed with successful excision of the tumour. Histological examination of the surgical specimen revealed an adenocarcinoma with abundant tumour necrosis; the tumour was composed of broad palisading glands, papillary formations, and dystrophic calcification (Figure 4). The immunohistochemical profile and morphological features were consistent with the diagnosis of metastatic adenocarcinoma of colorectal origin. The postoperative course was uneventful. The patient received postoperative whole brain radiotherapy and remained well with a good performance status.

## 3. Discussion

The calcification of brain metastases is extremely rare and can easily be misdiagnosed as a benign tumour [1]. Generally, the long periods required for the deposition of calcium result in the differential diagnosis of slow progressive benign cysts and tumours [2]. Despite the rarity of calcified brain metastases, correct identification of the pathology is vital towards tailoring an appropriate treatment regime.

The most common primary sites reported to produce calcified metastatic cerebral lesions include breast, lungs, ovaries, and colon [4]. The detection of calcified metastases is dependent on the imaging modality, with an incidence of 1.4% by plain skull radiographs and 3.5% by CT scanning [2, 5–7]. 2.3–4% of CRC patients are clinically diagnosed with brain metastasis [8, 9]. The most common site is the cerebellum, where 35–55% of CRC brain metastases are found [8–11]. Lesions in the frontal lobe are less common with a reported incidence of 23–33% [8, 12].

Our case demonstrates a solitary calcified frontal lobe nodule, which metastasised from CRC. Using simple arithmetic and known statistics we can appreciate why such a diagnosis can be difficult to determine. Assuming that 2.3% of CRC metastases to the brain and only 3.5% calcify, a rough estimate of only 0.08% of patients with CRC would have such radiological findings [6, 8].

Unlike other metastatic lesions that can be localised using positron emission tomography (PET) scan, brain metastasis proves to be difficult [13]. Magnetic resonance imaging has been established as a superior imaging modality for brain metastases [14]. Sánchez de Cos Escuín et al. reported that cranial MRI had a higher percentage of silent brain metastases diagnosed compared to cranial CT [14]. MRI also performed better than CT in the detection of multiple brain metastases and lesions smaller than 1 cm [14]. Magnetic resonance imaging therefore remains the gold standard for cerebral lesions [15].

Differentiating between metastatic cerebral lesions and benign intracranial neoplasms, such as a meningioma for example, is imperative to appropriate treatment planning. The best therapeutic approach to an asymptomatic benign lesion is conservative treatment with close follow-up to avoid surgical morbidity [16]. Our case highlights the importance of close follow-up of patients diagnosed with an asymptomatic cerebral lesion. Prior to commitment to conservative treatment the patient must be thoroughly investigated with MR imaging which is considered gold standard for cerebral lesions [17]. Once the clinical decision for conservative treatment has been made the following schedule for low grade tumour follow-up is recommended. MR imaging at a 3-month basis for the first 1 year followed by 6–12-month imaging for another 5 years, with progressively less frequent intervals for the years beyond [17].

The differential diagnoses for solitary calcified lesions depending on location include angiomas, meningiomas, and gliomas [18]. In comparison, the differential diagnoses for multiple calcified lesions include toxoplasmosis, postirradiation changes, tuberous sclerosis, tuberculoma, and other granulomatous diseases [18]. Amongst calcified metastatic lesions, the most commonly reported histological type is adenocarcinoma [4, 10].

McCutcheon reported a median survival of 1–3 months for patients with untreated CRC brain metastases [19]. The median survival for patients treated with surgery was greater at 9 months [19]. Mege et al. confirmed this benefit with surgical resection of brain metastases from colorectal carcinoma, increasing the median overall survival by up to 14 months [12]. Despite the lack of prognostic data specifically for calcified brain metastasis, the benefits of surgical treatment for brain metastases are evident. In the absence of systemic illness, the correct diagnosis enables prompt treatment with curative intent. In the context of disseminated disease, however, palliative treatment improves the patient's quality of life.

## 4. Conclusion

In conclusion, it is recommended that oncology patients should receive early brain scans even if asymptomatic, as it may lead to the early discovery of silent metastases leading to appropriate treatment, improved survival rates, and a heightened quality of life. Furthermore, the discovery of a single subcentimetric, nonenhancing calcific focus with no perilesional oedema in patients with a known oncology history must include metastases as a differential diagnosis, as although it is uncommon it is clinically significant.
